# Organising medication discontinuation: a qualitative study exploring the views of general practitioners toward discontinuing statins

**DOI:** 10.1186/s12913-016-1495-2

**Published:** 2016-07-07

**Authors:** Michael Nixon, Marius Brostrøm Kousgaard

**Affiliations:** Center for Healthy Ageing, Section of General Practice, Department of Public Health, Faculty of Health Sciences, University of Copenhagen, Copenhagen, Denmark; The Research Unit for General Practice and Section of General Practice, Department of Public Health, Faculty of Health Sciences, University of Copenhagen, Copenhagen, Denmark

## Abstract

**Background:**

Discontinuing medications is a complex decision making process and an important medical practice. It is a tool in reducing polypharmacy, reducing health system expenditure and improving patient quality of life. Few studies have looked at how general practitioners (GPs) discontinue a medication, in agreement with the patients, from a professional perspective. Three research questions were examined in this study: when does medication discontinuation occur in general practice, how is discontinuing medication handled in the GP’s practice and how do GPs make decisions about discontinuing medication?

**Methods:**

Twenty four GPs were interviewed using a maximum variation sample strategy. Participant observations were done in three general practices, for one day each, totalling approximately 30 consultations.

**Results:**

The results show that different discontinuation cues (related to the type of consultation, medical records and the patient) create situations of dissonance that can lead to the GP considering the option of discontinuation. We also show that there is a lot of ambiguity in situations of discontinuing and that some GPs trialled discontinuing as means of generating more information that could be used to deal with the ambiguity.

**Conclusions:**

We conclude that the practice of discontinuation should be conceptualised as a continually evaluative process and one that requires sustained reflection through a culture of systematically scheduled check-ups, routinely eliciting the patient’s experience of taking drugs and trialling discontinuation. Some policy recommendations are offered including supporting GPs with lists or handbooks that directly address discontinuation and by developing more person centred clinical guidelines that discuss discontinuation more explicitly.

## Background

Discontinuing medication is an important medical practice for a variety of reasons. It is a tool in reducing harms from taking drugs (e.g. side effects or drug-drug interactions), polypharmacy, reducing health system expenditure and improving evidence based medicine by discontinuing treatments that no longer have any evidence or indication [1, 2]. These challenges are compounded by an increasing prevalence of patients with several chronic diseases and several risk factors in high-income countries. These patients can easily have 20 indicated or recommended drugs on a medication list, and the challenge of prioritization and discontinuing medication, when necessary, becomes an important one [3]. Patients themselves have indicated they are interested in having more of their medication discontinued [4, 5]. There has been a growing interest in reducing the harms from taking drugs that has mainly focused on overdiagnosis and ‘the art of not doing’ [6–8]. We suggest that this interest in reducing harms caused by drugs would benefit from extending its focus to discontinuing medication.

Despite there being a tremendous focus on how to prescribe medication, there has been very little focus on the process of discontinuing medication [9, 10]. Patients are still being prescribed statins during end of life or palliative care, despite there being no benefit and some harm [11, 12]. Quantitative research has shown that discontinuation can be safe, that discontinuing multiple drugs is feasible and that it benefits patients and healthcare systems [1, 13, 14]. A feasibility study among elderly patients found that 4–5 drugs per patient could be discontinued, that 88 % of patients reported a global improvement in health and that only 2 % of patients needed to be restarted [14]. In another trial of discontinuation reductions in mortality, referrals to acute care and drug costs were reduced [1]. As a result of this research, there have been calls for a more proactive approach to discontinuing medication [15, 16].

However, there has been very little qualitative research that examines the mechanisms of discontinuing medication and how it is actually done in practice. Most of the qualitative research has primarily focused on the patient [17, 18], despite the general practitioner (GP) being an important actor. GPs (also known as family physicians) are the biggest prescribers of medication and are often those that have, willingly or unwillingly, the overall responsibility of a patient’s medication [3]. Therefore the process of discontinuing medication as a medical practice in primary care needs to be better understood.

Clinical decisions about discontinuation are often made in situations where the relation between causes and effects are unclear for individual patients. Such situations can be described as ambiguous, or a problem of too many interpretations that leaves the GP feeling uncertain about how to proceed [19]. For example, it may be unclear if the current treatment has any therapeutic value [20] or it may be unclear how to apply the clinical guidelines to a specific patient [3]. Whilst characteristics of ambiguous discontinuing situations have been described, this study is interested in how GPs respond to such situations as little is currently known about it.

When viewing discontinuing medication as a process it is important to understand where it occurs, how it is organised and the role of ambiguity. Thus the research questions in this study are:

1. When does medication discontinuation occur in general practice?
2. How is discontinuing medication handled in the GP’s practice?
3. How do GPs make decisions about discontinuing medication?

Throughout the study we use the term ‘discontinuing’ rather than ‘deprescribing’ because it is a richer analytical description of the process. For example, patients cannot deprescribe their medication, but they can discontinue their medication. So even though this study’s focus is the professional, it is still important to allow for a patient’s agency in the process.

### The case of statins

Statins are an ideal case for examining the practice of discontinuing medication. Statins are an institutionally recommended drug [21] and the most prescribed drug in Europe [22, 23]. It is therefore a drug that affects a large number of people, even though its usage in primary prevention has been criticized, and its side effects have been underestimated [24]. Statins are an example of risk reducing medication that are recommended in guidelines and often given over long periods of time, and which are difficult for GPs to discontinue [3, 25]. Guidelines in Denmark recommend a statin when the patient‘s cholesterol is over 5 millimoles/litre total cholesterol for persons with previous cardiovascular events, and this acts as a kind of ‘cut-off’ for GPs. For persons without previous cardiovascular events, the HEART SCORE risk card is one of the main tools used to decide if they should start a statin. It consists of a colour-coded chart with the colours of red and yellow and green to indicate what a person’s risk is and whether they should be prescribed a statin. Because of how relevant statins are to many people and because they are a recommended drug that is difficult for the GP to discontinue, they were chosen as the case for examining discontinuing medication.

## Methods

Two data collection methods were employed in this study: semi-structured interviews and participant observations. 24 GPs were selected for interviews using a sampling strategy of maximum variation [26]. The sampling criteria were length of practice, gender, size of the practice, industry sponsorship (yes/no) and geographical region. A summary of informant characteristics is shown in Table 1. Having surveyed recent qualitative studies of GPs experiences with medication issues, where sample sizes varied from 7 to 29 with most of these being focus group studies [3, 27–29] we considered 24 informants to be an appropriate sample size for an explorative study like this [30]. Further, after having performed the interviews data-saturation was apparent (although we cannot completely rule out that that new themes might have been generated by performing more interviews and observations).

**Table 1:** Characteristics of the informants

ID	Years of experience	Practice organisation	Geographical region
GP 1	25	Group	Capital
GP 2	18	Group	Capital
GP 3	16	Group	Capital
GP 4	17	Solo	Capital
GP 5	16	Solo	Capital
GP 6	20	Solo	Capital
GP 7	15	Group	South Denmark
GP 8	15	Group	South Denmark
GP 9	9	Solo	Zealand
GP 10	24	Group	Capital
GP 11	23	Group	Capital
GP 12	22	Solo	Capital
GP 13	2	Group	Capital
GP 14	18	Group	Zealand
GP 15	20	Group	North Jutland
GP 16	3	Group	South Denmark
GP 17	14	Group	South Denmark
GP 18	26	Solo	Capital
GP 19	0	Group	South Denmark
GP 20	18	Group	South Denmark
GP 21	18	Group	South Denmark
GP 22	15	Group	Capital
GP 23	0	Group	North Jutland
GP 24	22	Group	North Jutland

**Table 2:** Examples of cues that create dissonance

Patient based cues	Record based cues
Side effects (muscle pain, depression)	High number of drugs
High drug burden	No history of CVD
Patient wants to stop	Hospital discharge
Patient concerns from media	Blood test results will 70+ years of age

Interviews were on average one hour, recorded and transcribed afterwards. A pilot study of five interviews was done to develop and refine the interview guide. The interviews began by asking the GPs for recent examples of discontinuing statins so that they could be as concrete as possible about their reflections and action. If they could not give any recent examples, the themes from the next part of the interview guide were used. These included how the GPs kept themselves up-to-date with knowledge of prescribing and discontinuing and how they organised discontinuation in their practice.

Participant observations were done in three general practices. Each practice was observed for one day, totalling approximately 30 consultations. The observation days were chosen beforehand together with the GP so that these days were more likely to contain consultations involving discussions about medication. The GPs in the observed practices were interviewed beforehand, and in between consultations the GPs often explained their thought processes and their considerations of the preceding consultation. Notes were taken during the observations, and after the observations supplementary notes were recorded together with reflections on the observations of the day [31]. Participant observations generated data on the process of discontinuation and how it unfolded over time, and thus it complemented the interview data on the GPs’ reasoning and attitudes on discontinuation.

The method of analysis used was the Gioia method [32]. Here the data was coded inductively and two levels of data were developed: first order concepts and second order themes. The first order concepts generated were the initial codes and categories that closely reflect the original informant vernacular. Second order themes were developed by introducing the theoretical concepts of cues and attention to the first order concepts, so that analytical similarities and differences were grouped together and the themes offered a more theoretical perspective on the data. The study was inspired by sensemaking theory, a process based perspective that focuses on how people create meanings that shape their actions (and vice versa) [19, 33]. So the focus was on individuals rather than institutions. During the process of data collection and analysis, the two concepts of cues and ambiguity emerged as the most relevant theoretical concepts of sensemaking, and so were introduced to the data to further refine the findings. In this sense, the theoretical concepts acted as a ‘sensitising instrument’ that increased our sensitivity to analytically relevant data that otherwise may have been missed had if strictly inductive approach was used [34].

During the analysis we also looked for things that surprised or puzzled us in the data. For example, when GPs were asked about what prompted them to consider discontinuation, they often quickly and easily replied because of side effects. We could have stopped there and labelled ‘side-effects’ as cues, but we sensed that the ease with which they said it hid a lot of the work that had to go into generating this stable and medically accepted label ‘side effects’. Hence we wanted to find out more about how these ‘side effects’ arose as an issue in the first place and this eventually led to one of the study’s key themes (of patient and record cues). During the analytical process, the techniques of negative cases, constant comparisons and peer debriefing were used to improve the quality of the analysis [32, 35].

## Results

Only four of the GPs stated that they regularly discontinued medication. The majority of the respondents reported that discontinuation was rarely done, and as a specific practice they did not consider it to be very organized. As one GP noted:

*We have always been bad at discontinuing something I think. I’ve, you know, been a doctor for 14 years now and I think that we become better at discontinuing some of it, but we often talk about that we don’t really do enough about it [discontinuing]. [GP17; p.3]*

This description of discontinuing medication as something often talked about, but rarely done, was frequent among GPs.

There were at least two reasons for why discontinuing medication was rarely done. First, the routine of prescribing was so strong that a concerted effort is needed to even raise the possibility of discontinuing medication:

*It’s clear that, when discontinuing medication, as soon as it’s in the system, you don’t stop any medicine, unless you yourself implement some systems that flag it up [as an issue to discuss]. [GP20; p.6]*

And the GP sometimes forgot to discuss the possibility of discontinuation with the patients, especially if the patient did not mention it:

*Sometimes you continue without thinking about it. The patient doesn’t come in and ask [for discontinuation]. They ask for the same medication. And you think they benefit from it. [GP6; p.4]*

Second, it was difficult for the GPs to identify the right time to discuss discontinuation with the patient:

*Because you can say she has been in the clinic for a lot of other things in between [the first prescription and the current check up] and you don’t sit and think, her cholesterol is looking good and therefore I suddenly want to look 12 years back in the journal to find out if the original indication was justified or not. [GP23; p.8]*

Thus, a mixture of a strong culture of prescribing and the difficulty of identifying relevant patients for discontinuing medication led to a sense of discontinuing medication being challenging to do.

### The role of cues and dissonance for discontinuing medication

Although deliberations about discontinuing a patient’s medication could come about in many different ways, such deliberations tended to occur more frequently in certain situations; e.g. consultations that involved a check up (annual chronic disease check up, cholesterol check up, nursing home check up) or consultations with new patients (from another practice). Such situations were characterized by being more likely to generate what can be called cues of discontinuation. A cue of discontinuation is something that attracts the GP’s attention toward the possibility of discontinuing medication. If enough attention is drawn to the possibility of discontinuation, then a dissonance situation is created, where a question mark is put by the necessity of the prescription.

Patient based cues are cues emerging from the patient that the GP would not be able to pick up on his/her own, without the patient. Examples include: a patient complains over side effects (muscle pain, depression) or a high drug burden, a patient makes an explicit wish to stop medication, a patient shows concern over media reports about problems with specific drugs:

*I have discussed it as recently as today with a patient that takes a lot of medicine, who has also had some different symptoms and she was very tired of taking so much medicine. She didn’t ask about statins specifically, to Simvastatin, which she took, but the idea of reducing her statin was a good thing for her. Also because she had so many strange things with her body, that may actually be related to statins. [GP22; p.1]*

Record based cues are data related cues that can be extracted by the GP without the patient being present. Examples include blood test results, patients’ age, number of drugs prescribed, history of CVD. For example, one GP said:

*A healthy woman aged 87 came to me, she’s on a statin, and she’s changed her GP [to me]. She gets simvastatin and she has no diabetes, no ischemic heart disease and a cholesterol score of 5,2 [the threshold is 5]. [GP10; p.9]*

Here a variety of record based cues, accessible from records independent of the patient, prompt the GP to consider discontinuing the statin.

Some cues might singlehandedly trigger considerations about discontinuing medication, e.g. patient changes GP practice, whereas others might act in conjunction with other cues, e.g. a patient mentions concern about side effects and the GP then looks at the record and sees no previous history of cardiovascular disease (Table 2).

### Organising the discontinuation of medication

Despite the rarity and difficulty of discontinuing medication, there were some GPs who attempted to organise discontinuation more systematically, and they did so in two ways. The first was to actively schedule check-ups and monitoring for a variety of their patients with a need for reviewing their medication situation. GP 9’s strategies included visits after hospital discharge and planning hospital admissions (when possible):

*I mean, when a patient is discharged from the hospital, then I get a discharge summary and then within a week I actually visit them. I mean, if I choose to come within a week, then I’m able to stop most of it [unnecessary medication] before the home nurse starts giving it for a long time. So that way I’ve got a grip on it. And I also think it’s pretty fun. [GP9; p.8]*

Here the GP is proactive in identifying the patient’s medication needs and examining the necessity of any new prescriptions. No special reimbursement was available to the GP for this extra work in term of identifying and visiting relevant patients (though new tariffs have since been introduced for check-ups with vulnerable patients). However, the GP saw the process as a stimulating professional challenge, which presumably contributed to his willingness to perform these systematic checks.

The importance of having this routine systematisation of monitoring was emphasized by another GP:

*It has something to do with the routines that we have [in the practice], where we have a clear agreement between us, that such and such prescription renewal MUST come in for a check-up or they get a bit now, and know that soon in the future they have to come in for a check-up. This is for select areas, where we know there are problems. [GP14; p.9]*

If a practice does not proactively invite the patient in for a check-up (and often the responsibility of represcription lies with the secretary) then it is difficult to identify and monitor patients that may benefit from discontinuing medication. This shows the important role of other actors besides GPs and patients, as well as the level of organisation needed to systematically consider discontinuation.

Prescriptions are supposed to be renewed every three to four months in an effort to continually assess the drug’s relevance for the patient. However, renewing the represcriptions often occurs without the patient. So despite there being a theoretical control of medications one GP noted that the reality is not always as such:

*They need to, you know, have their prescription renewed, so you get a hold of them at some point or another. I mean, people that get statins need to be checked. In principle they should be checked every fourth month, I think it says in the guidance, for their liver function. So they come to the check-up of the statin… But, I mean there are also a lot that continue to take them [statins] for years without being checked [GP24; p.16]*

In other words, organising the discontinuation of medication by scheduling check-ups is necessary, but not sufficient in itself. It also requires social interaction - the patient must be there.

The second way of organising discontinuation was to elicit more explicitly the patient’s experience of taking the statin, as opposed to relying on the patient to self-report any important information.

*It makes no sense, if you… can’t lift yourself and sit down, because you’re on a statin. It is incredibly different how people react, so it is so, so important that you follow up, especially after you’ve started something, to see how big a burden it might be. [GP1; p14]*

A patient’s negative experience with taking statins was often an important factor in the GP’s decision to discontinue. However it was often difficult to predict which patients would suffer from harms when taking statins or which patients would experience their burden of drug treatment as ‘too high’. Therefore the patient’s experience of taking the drug was important information for the GP after treatment started. A difference between GPs was observed in how actively the patient’s experience was elicited. Some GPs preferred to rely on the patient to schedule their consultations after experiencing harms, or to report it themselves at check-ups. Other GPs were more systematic in following up and eliciting patient’s experience with medication, including the effects of individual drugs, the overall drug burden and patient’s fears or concerns about taking a drug.

### Responding to ambiguity

Occasionally there were instances where discontinuing medication was the obvious thing to do and where the GP would simply discontinue the statin. For example, one GP had a new patient from another practice and saw her for a second consultation. She took a lot of drugs and they had been discussing her experiences with this. She felt the burden was high, and that there were side effects from the statins. She had no history of cardiovascular disease and her cholesterol scores were not high. The GP commented:

*I mean in my world I would discontinue straightaway and see what happened. She has as I said not had any infarctions and so on. So it will be an obvious thing for me to do, to discontinue that. But then she is an easy case. [GP22; p.3]*

As the final sentence indicates, such clear-cut situations were rare and the majority of decisions around discontinuing medication involved responding to some level of ambiguity.

A GP elaborates on the challenges of negotiating conflicting sources of information and evaluating the effects of the treatment on the patient:

*It is, you know, like a jigsaw puzzle because I actually think that it is really hard [knowing when it is best to discontinue], because I’ve taken it up a lot of times with many different patients, exactly the issue of an isolated raised cholesterol level, and when you should begin treatment. And there’s no-one that agrees, is my sense… it’s a personal call, and yeah, we know about all that risk stuff, and that it is not completely OK [to use SCORE risk charts for persons over 65] … but we don’t have anything better and… [sigh].. I just think it’s hard. I don’t think it’s as black and white [as guidelines say], because who says that that is a good solution for the individual patient? [GP19; p.13]*

For the GP, the lack of agreement and conflicting information about when to prescribe or discontinue the drug, made it hard to decide with confidence what was most appropriate for the patient. The GP noted that tools like the SCORE heart risk colour chart was often used to reduce the ambiguity with elderly patients, even though she knew it was not appropriate for people over 65. For the GP, these difficulties with determining the best course of action showed that the clinical reality was more ambiguous than suggested by the guidelines.

A common response was to see this ambiguity as a reason to continue the treatment. Because of the risk of an adverse event happening after discontinuing medication, the safer option was to simply continue prescribing, thinking that it was better to have tried giving treatment than not giving treatment, if something went wrong. There was a fear that:

*…you overlook something or make a mistake, and so you’d better do as they say in all the recommendations [guidelines]. I know so many that… I mean if there is a guideline, and it says that we are in the yellow field here, and that treatment should get started, then you start [prescribing the drug]. I mean in that flowchart [for cardiovascular risk] it can be yellow or red. If you are in the yellow field, then you start [treatment], because then you at least haven’t done something wrong. Because if they [the patient] had a blood clot and you haven’t prescribed anything, then you can’t [protect yourself and] claim that they were at least being prescribed [the drug]. [GP19; p.25]*

In contrast, there were GPs that saw ambiguity as a reason trying out discontinuation for a short period followed by a check-up consultation. One GP elaborated on the advantages of pausing, rather than discontinuing straight away:

*So I say to the patient, I think we should just have a break, and making use of a break to see if there are any changes. But then it means that there is usually a new decision after the break, you have to decide whether to continue the drug again or whether it should be discontinued. So in that way, a break amounts to two decisions, where discontinuation demands just one, but that decision is just somewhat bigger. So a break is a good way to start discontinuing you could say. [GP20; p.11]*

Trialling was a way of practising medication discontinuation without psychologically committing oneself indefinitely to the process. Rather than one, larger decision it became two smaller decisions. A pause of one to 6 months generated more information (e.g. does the patient’s muscle pain disappear or does the cholesterol level shoot up) and provided an opportunity to revisit the decision and reflect on its appropriateness. Thus, trialling allowed GPs to generate more cues if they felt they had a good reason to consider discontinuation, but not enough information to be certain. In sum, it was an acceptable way of trying out discontinuing medication without being ‘committed’ to the action, because it was just a ‘trial’.

## Discussion

This study contributes to the literature on discontinuing medication by exploring the perspectives of GPs. Generally, discontinuing institutionally recommended drugs, in this case statins, was found to be rare, but there were some GPs who discontinued systematically, despite the ambiguity surrounding the outcome. Nearly all instances of reported discontinuing had elements of dissonance. Those GPs that discontinued systematically created situations of dissonance deliberately. In doing so, they created a deeper culture of reflecting on the medication their patients take.

Creating this culture through situations of dissonance was done in at least two ways. Firstly, by systematically arranging a variety of different check-up consultations such as visits after hospital discharges or medication reviews of elderly home patients. Secondly by actively eliciting the patient’s experience of taking drugs, including the effects of individual drugs, the overall drug burden and patient’s concerns about taking specific drugs. The consequence of using both strategies was to produce more discontinuation cues, which in turn increased the likelihood of enacting discontinuation. This study complements previous research by showing the importance of using patient’s experiences in decisions to discontinue [20]. The study also shows that discontinuing medication is more likely to occur when organised proactively, rather than reactively, e.g. after a patient suffers from some kind of harm.

The reasons the GPs gave for discontinuing medication echo the reasons physicians give for abstaining from guideline recommended drugs [27]. For example the GPs sometimes choose to discontinue/abstain from treatment because of concerns about the harms of the drug, or because of problems with a high burden of treatment [27, 36]. We suggest that to frame the choice of GPs to discontinue guideline recommended drugs as an inability to follow guidelines with negative normative consequences or as ‘inertia’ is an unproductive one. Instead, a more fruitful approach could be to understand discontinuing medication as clinically relevant care and to acknowledge and support it as such. As one study on clinical inertia concluded, ‘much inaction may be clinically appropriate care’ [37]. Instead of castigating GPs for a lack of action, more effort should be directed towards understanding why GPs choose not prescribe a drug or to discontinue a drug.

Another interesting point from this study is how some GPs saw ambiguous situations as a reason *to* act and trial discontinuing with the patient over a period of time, rather than a reason *not* to act. This strategy of trialling to address the ambiguity of discontinuation has the effect of producing more cues for the GPs to use in their decision-making. This supports Weick’s [19] claim that reducing ambiguity requires access to more varied cues and the ability to argue for one interpretation over competing alternatives. In the case of discontinuing, GPs can use the information produced by the trial, e.g. if the harms disappear or not, to decide together with the patient if they should confirm or abort the ‘half-decision’ to discontinue.

Our findings suggest that the discontinuation of medication is a continually evaluative process (e.g. trialling, restarting at a later date) that depends on the patient’s situation. This approach is in line with the literature around situated judgment [9], that decision making draws on different kinds of justifications and is not solely determined by the amount of information available [38]. What these results show is how important it is that GPs feel more certain about the choice of discontinuing and that they can argue for that choice. So GPs can never act with complete certainty and discontinuing will remain an iterative process requiring a variety of strategies to deal with ambiguity.

### Strengths and weaknesses

One of the main strengths of this study is that different types of data were collected – both interviews and participant observations with GPs were used in the analysis. This allowed for themes to be more comprehensively developed and crosschecked. For example, the retrospective accounts of interviews were compensated for by the current accounts of participant observations. The second strength of the study is the explicit focus on GP reflections towards discontinuing medication. Very few studies have investigated GP attitudes to discontinuation and currently only one has explicitly done so [3], so this study offers novel insights into the professional process of discontinuing medication.

The study also has some weaknesses. Only one drug, statins, was examined, and it is an example of a risk-reducing drug that is prescribed on the basis of risk, and not symptoms. Future research could focus on discontinuing symptomatic medicine, including addictive drugs like SSRIs for depression, as issues may be different for those drugs. The study setting was in Denmark, and the findings may apply differently to different countries, especially those without general practitioners in a primary health care system, e.g. the USA. Finally we note two weaknesses regarding our data collection. The participant observation data could have been strengthened by observing all of the practices of the interviewed GPs, and not just three practices (accounting for 5 of the GPs interviewed). This would have allowed a more extensive data analysis. Second, the participants were informed of the study focus on discontinuation beforehand. This was done so that they could bring stories of discontinuation to the interview (a pilot showed that it was difficult for them to spontaneously relate such stories) and suggest relevant patient consultations to be observed. Thus it is possible that the participants may have overstated their interest and competences concerning the topic at hand. However, and contrary to our expectations, the GPs were harsher on themselves in interviews than when they were observed in practice. So there was little evidence of them flattering themselves in their accounts of discontinuation, even though they were aware of the study’s topic.

### Implications for policy and practice

The results of the study suggest that creating a culture of reflecting on patients’ medication during consultations, eliciting patient experiences of taking drugs, and trialling discontinuation can inform future policy and practice. Firstly, GPs can be encouraged to systematically schedule regular check-ups for relevant patients, especially those patients that cross healthcare sectors, like hospitals or nursing homes. Examples of such consultations include regular follow-up visits after hospital discharge for people over 65 or biannual visits to nursing homes. However, performing one type of consultation, e.g. medication reviews for people over 65, may be insufficient in creating a stronger culture of reflecting on patients’ medication. Such a culture could be supported by pro-actively scheduling, with the help of receptionists [39], a variety of different types of check-ups, so that GPs can systematically create situations where they are more likely to consider discontinuing with the patient. During these consultations, GPs can be encouraged to systematically elicit patient experience of taking the drug, e.g. potential harms, fears or a high drug burden, in order to facilitate shared decision making. The suggestion of supporting GPs in eliciting patient experiences and preferences is supported in other discontinuing medication literature [3, 40].

GPs currently try to reduce ambiguity on a small scale by individually trialling discontinuation. This process of reducing ambiguity can be supported on a larger scale, for example, by developing discontinuation lists or handbooks [41, 42]. One of the difficulties with discontinuing statins is that they are institutionally recommended in clinical guidelines, so more information is needed on how and when to discontinue institutionally recommended drugs [15]. Part of the problem is that guidelines tend to focus on diseases and treatment targets, rather than the person taking the medication [43]. So developing more person centred guidelines that prioritise the person over the disease with dedicated sections addressing discontinuing medication could support GPs by better reflecting their clinical realities [25].

## Conclusion

The process of discontinuing medication was found to be a complex and ambiguous process for GPs in general practice. Discontinuation cues created situations of dissonance between the option of discontinuing or continuing the patient’s medication. If the high level of ambiguity meant the GP was in doubt about whether to discontinue or not, the strategy of trialling discontinuation for a period of time to generate more information before committing to the decision with the patient could be used. Suggestions for the future policy are centred on sustained reflection around discontinuing medication, by systematically flagging it up through a culture of systematically scheduled check-ups and generating information by explicitly and regularly eliciting the patient’s experience of taking drugs. The usefulness of these options needs to be explored more thoroughly if discontinuing medication is to reduce avoidable drug-related harms to patients and improve their quality of life.

## Abbreviations

CVD, cardiovascular disease; GP, general practitioner.

## References

[1] Garfinkel D, Zur-Gil S, Ben-Israel J (2007). The war against polypharmacy: a new cost-effective geriatric-palliative approach for improving drug therapy in disabled elderly people. Isr Med Assoc J.

[2] Zed PJ (2011). Adverse Drug Events and Hospital Pharmacy Practice : Thinking Outside Our Box. Can J Hosp Pharm.

[3] Schuling J, Gebben H, Veehof LJG, Haaijer-Ruskamp FM. Deprescribing medication in very elderly patients with multimorbidity: the view of Dutch GPs. A qualitative study. BMC Fam Pract. 2012;13:56. 10.1186/1471-2296-13-56 PMC3391990 22697490

[4] Straand J, Sandvik H (2001). Stopping long-term drug therapy in general practice. How well do physicians and patients agree?. Fam Pract.

[5] Reeve E, Wiese MD, Hendrix I, Roberts MS, Shakib S (2013). People’s attitudes, beliefs, and experiences regarding polypharmacy and willingness to Deprescribe. J Am Geriatr Soc.

[6] Grady D, Redberg R (2010). Less is more: how less health care can result in better health. Arch Intern Med.

[7] Moynihan R, Doust J, Henry D (2012). Preventing overdiagnosis: how to stop harming the healthy. Br Med J.

[8] Cassel C, Guest J (2012). Choosing wisely: helping physicians and patients make smart decisions about their care. J Am Med Assoc.

[9] Clinch M, Benson J (2013). Making information “relevant”: general practitioner judgments and the production of patient involvement. Soc Sci Med.

[10] Alldred DP (2014). Deprescribing: a brave new word?. Int J Pharm Pract.

[11] Kutner JS, Blatchford PJ, Taylor DH, Ritchie CS, Bull JH, Fairclough DL, Hanson LC, LeBlanc TW, Samsa GP, Wolf S, Aziz NM, Currow DC, Ferrell B, Wagner-Johnston N, Zafar SY, Cleary JF, Dev S, Goode PS, Kamal AH, Kassner C, Kvale E a., McCallum JG, Ogunseitan AB, Pantilat SZ, Portenoy RK, Prince-Paul M, Sloan J a., Swetz KM, Von Gunten CF, Abernethy AP. Safety and Benefit of Discontinuing Statin Therapy in the Setting of Advanced, Life-Limiting Illness. JAMA Intern Med. 2015;175:691–700. 10.1001/jamainternmed.2015.0289 PMC4618294 25798575

[12] Turner JP, Shakib S, Singhal N, Hogan-Doran J, Prowse R, Johns S, Thynne T, Bell JS (2014). Statin Use and Pain in Older People with Cancer: A Cross-Sectional Study. J Am Geriatr Soc.

[13] Iyer S, Naganathan V, McLachlan AJ, Le Couteur DG (2008). Medication withdrawal trials in people aged 65 years and older: a systematic review. Drugs Aging.

[14] Garfinkel D, Mangin D (2010). Feasibility study of a systematic approach for discontinuation of multiple medications in older adults: addressing polypharmacy. Arch Intern Med.

[15] Scott I, Gray L, Martin J, Pillans P, Mitchell C (2012). Deciding when to stop: towards evidence-based deprescribing of drugs in older populations. Evid Based Med.

[16] Scott I, Hilmer S, Reeve E, Potter K, Le Couteur D, Rigby D, Gnjidic D, Del Mar CB, Roughead EE, Page A, Jansen J, Martin JH (2015). Reducing Inappropriate Polypharmacy. JAMA Intern Med.

[17] Reeve E, To J, Hendrix I, Shakib S, Roberts MS, Wiese MD (2013). Patient barriers to and enablers of deprescribing: a systematic review. Drugs Aging.

[18] Reeve E, Shakib S, Hendrix I, Roberts MS, Wiese MD (2014). Review of deprescribing processes and development of an evidence based, patient-centred deprescribing process. Br J Clin Pharmacol.

[19] Weick K (1995). Sensemaking in Organisations.

[20] Ostini R, Jackson C, Hegney D, Tett S (2011). How Is Medication Prescribing Ceased? A systematic review. Med Care.

[21] Goff DC, Lloyd-Jones DM, Bennett G, Coady S, D’Agostino RB, Gibbons R, Greenland P, Lackland DT, Levy D, O’Donnell CJ, Robinson JG, Schwartz JS, Shero ST, Smith SC, Sorlie P, Stone NJ, Wilson PWF (2014). 2013 ACC/AHA Guideline on the Assessment of Cardiovascular Risk: A Report of the American College of Cardiology/American Heart Association Task Force on Practice Guidelines. Circulation.

[22] Walley T, Folino-Gallo P, Stephens P, Van Ganse E (2005). Trends in prescribing and utilization of statins and other lipid lowering drugs across Europe 1997–2003. Br J Clin Pharmacol.

[23] Parker BA, Thompson PD (2013). Effect of Statins on Skeletal Muscle: Exercise, Myopathy, and Muscle Outcomes. Exerc Sport Sci Rev.

[24] Ray KK, Seshasai SRK, Erqou S, Sever P, Jukema JW, Ford I, Sattar N (2010). Statins and All-Cause Mortality in High-Risk Primary Prevention. Arch Intern Med.

[25] Mangin D, Heath I, Jamoulle M (2012). Beyond diagnosis: rising to the multimorbidity challenge. Br Med J.

[26] Dahlgren L, Emmelin M, Winkvist A (2007). Qualitative Methodology for International Public Health.

[27] Elisabeth A, Denig P, van Vliet T, Dekker JH (2009). Reasons of general practitioners for not prescribing lipid-lowering medication to patients with diabetes: a qualitative study. BMC Fam Pract.

[28] Dekker F, Knuistingh Neven A, Andriesse B, Kernick D, Reis R, Ferrari MD, Assendelft WJ (2012). Prophylactic treatment of migraine; the patient’s view, a qualitative study. BMC Fam Pract.

[29] Kirkegaard P, Risor MB, Edwards A, Junge AG, Thomsen JL (2012). Speaking of risk, managing uncertainty: Decision-making about cholesterol-reducing treatment in general practice. Qual Prim Care.

[30] Marshall B, Cardon P, Poddar A, Fontenot R (2013). Does sample size matter in qualitative research? A review of qualitative interviews in is research. J Comput Inf Syst.

[31] Emerson R, Fretz R, Shaw L (1995). Writing Ethnographic Fieldnotes.

[32] Gioia D, Corley K, Hamilton A (2012). Seeking Qualitative Rigor in Inductive Research: Notes on the Gioia Methodology. Organ Res Methods.

[33] Weick K, Sutcliffe KM, Obstfeld D (2005). Organizing and the Process of Sensemaking. Organ Sci.

[34] Vaughan D, Ragin C, Becker H (1992). Theory Elaboration: the heuristics of case analysis. What is a case? Exploring the Foundations of Social Inquiry.

[35] Tracy SJ (2010). Qualitative Quality: Eight “Big-Tent” Criteria for Excellent Qualitative Research. Qual Inq.

[36] Steinman MA, Patil S, Kamat P, Peterson C, Knight SJ (2010). A taxonomy of reasons for not prescribing guideline-recommended medications for patients with heart failure. Am J Geriatr Pharmacother.

[37] Safford MM, Shewchuk R, Qu H, Williams JH, Estrada CA, Ovalle F, Allison JJ (2007). Reasons for not intensifying medications: differentiating “clinical inertia” from appropriate care. J Gen Intern Med.

[38] Moreira T (2005). Diversity in clinical guidelines: the role of repertoires of evaluation. Soc Sci Med.

[39] Swinglehurst D, Greenhalgh T, Russell J, Myall M (2011). Receptionist input to quality and safety in repeat prescribing in UK general practice: ethnographic case study. Br Med J.

[40] Ostini R, Hegney D, Jackson C, Tett S (2012). Knowing How to Stop : Ceasing Prescribing. J Manag Care Pharm.

[41] Tilyard M (2010). A Practical Guide to Stopping Medicines in Older People. BPJ.

[42] Lundgren C (2010). Phase Out [Fas Ut].

[43] Greenhalgh T, Howick J, Maskrey N (2014). Evidence based medicine: a movement in crisis?. Br Med J.

[44] Act on Research Ethics Review of Health Research Projects (Section 2, §1 + Section 14, §2). http://www.dnvk.dk/English/actonabiomedicalresearch.aspx. Accessed 23 June 2016.

